# Enhancing Shelf Life and Nutritional Quality of Lamb Burgers with *Brassica* By-Products: A Synergistic Approach Using High Hydrostatic Pressure

**DOI:** 10.3390/foods14040594

**Published:** 2025-02-11

**Authors:** Matilde D’Arrigo, Jonathan Delgado-Adámez, Jesús J. García-Parra, Irene Palacios, Montaña López-Parra, Ana Isabel Andrés, María Rosario Ramírez-Bernabé

**Affiliations:** 1 Centro de Investigaciones Científicas y Tecnológicas de Extremadura (CICYTEX), Instituto Tecnológico Agroalimentario (INTAEX), 06187 Badajoz, Spain; matilde.darrigo@juntaex.es (M.D.); jonathan.delgado@juntaex.es (J.D.-A.); jesusjavier.garcia@juntaex.es (J.J.G.-P.); irenepalaciosromero@gmail.com (I.P.); montana.lopez@juntaex.es (M.L.-P.); 2 Food Technology Department, School of Agricultural Engineering, University of Extremadura, 06007 Badajoz, Spain; aiandres@unex.es

**Keywords:** vegetable by-products, high hydrostatic pressure, broccoli, cauliflower, lamb, shelf life

## Abstract

This study examines the effects of incorporating broccoli and cauliflower by-products (leaves, stems and inflorescences) like puree ingredients and applying high hydrostatic pressure (HHP) treatment on the quality, safety, and shelf life of lamb burgers. Broccoli and cauliflower by-products were valorized like rich bioactive ingredients, especially in phenol compounds. The valorized ingredients were added to lamb burgers (5% *w*/*w*), and 120 burgers were produced for the experiment: three formulations (lamb, lamb with broccoli, and lamb with cauliflower) × four pressure treatments (untreated, 400 MPa, 500 MPa, 600 MPa) × five replicates per formulation and pressure treatment × two storage times (day 1 and day 14). The interactions between composition and pressure were also investigated. The results indicated that while *Brassica* by-products contributed to slight changes in moisture content and fatty acid composition, they did not independently provide strong antimicrobial effects, likely due to their high moisture content and minimal impact on pH reduction. However, combining these ingredients with HHP treatment (600 MPa for 60 s) significantly improved microbial stability. HHP treatment effectively reduced microbial counts, which were maintained during refrigerated storage, supporting its role as a valuable non-thermal intervention for enhancing meat safety. In terms of oxidative stability, the inclusion of *Brassica* ingredients, particularly with HHP, reduced lipid (TBA-RS ≤ 1.47 MDA mg kg^−1^) and protein oxidation (≤5.05 Nmol mg^−1^ proteins) over time, thereby enhancing product stability during storage. Sensory evaluation and affective testing revealed no significant differences in appearance, odor, taste, texture, or overall acceptability between treated and untreated samples, with high acceptance scores. This suggests that HHP treatment, in combination with *Brassica* by-products, can improve safety and oxidative stability without compromising the sensory quality of meat products. Overall, this study presents a sustainable and effective approach for producing high-quality and safe meat products with extended shelf life.

## 1. Introduction

It is projected that, by 2030, the global consumption of lamb will remain a niche market in some countries and become a major component of the diet in many others. This represents a 6% global increase in lamb consumption. Currently, the per capita consumption of sheep meat is comparable in both developing and developed countries. Additionally, lamb production is considered sustainable, as these animals can withstand adverse climatic conditions [1]. A popular use of lamb meat is in burgers, which are known worldwide for their ease of preparation, low cost, and pleasant flavor [2]. Sheep meat offers valuable physicochemical and nutritional properties, including high levels of protein, minerals such as iron and zinc, B-complex vitamins, and a substantial amount of unsaturated fatty acids. Some of these fatty acids help reduce LDL cholesterol and lower the risk of coronary heart disease [3]. Brassica vegetables are widely consumed and have significant economic importance, with well-known health benefits and disease prevention properties. Broccoli and cauliflower (*Brassica oleracea* var. *botrytis* and *var. italica*) provide essential nutrients such as vitamins, minerals, and fiber, as well as a complex mixture of phytochemicals, including glucosinolates and phenolic compounds [4]. These phytochemicals exhibit antimicrobial, anti-inflammatory, and anti-hypertensive activities. Among the phytochemicals with antioxidant properties, phenolic compounds are one of the most important groups. Moreover, the use of broccoli and cauliflower by-products (leaves, stems, and inflorescences) as rich sources of functional ingredients adds value while reducing waste and environmental impact [5,6,7]. The wide range of phenolic contents depends on the species and parts of the *Brassica* plant [6].

A wide variety of chemical, biological, thermal, and non-thermal technologies exist to preserve and protect food against microbiological growth and deterioration. HHP is one of the most widely used non-thermal technologies in commercial applications. It can inactivate foodborne microorganisms and enzymes; while covalent bonds remain intact, weaker bonds such as hydrogen and hydrophobic bonds can be irreversibly altered [8]. HHP treatment is an advanced and effective technology for developing meat and new meat products with relatively low energy consumption [9,10,11,12]. The treatment applies pressure ranging from 300 to 600 MPa for less than 10 min, using water immersion at room or refrigeration temperature. HHP treatment has been approved by the Food and Drug Administration (FDA), United States Department of Agriculture (USDA), European Food Safety Authority (EFSA), Health Canada, and other regulatory agencies globally [13].

The pH and water activity are key factors in effectively inactivating foodborne microorganisms during HHP treatment. A lower pH (<4.5) and lower water activity (a_w_ < 0.94) significantly enhance inactivation, making sublethal injured cells more susceptible to HHP [14]. By combining these parameters with other technologies, HHP can effectively overcome the resistance of bacterial endospores and food enzymes. HHP treatment can influence the texture, structure, lipid oxidation, and color of meat and meat products [14,15], creating opportunities to develop innovative products or improve the functionality of certain ingredients. Therefore, the application of HHP technology in the meat sector requires optimizing treatment conditions for each product and commercial presentation, often in combination with new packaging systems, natural antimicrobial agents, enzymatic formulations, etc. Recent studies have also evaluated the use of HHP to valorize agri-food by-products, demonstrating extended shelf life and enhanced product stability.

HHP extended the shelf life of marinated beef by delaying the growth of spoilage microorganisms and reducing safety risks associated with *Salmonella* sp. and *L. monocytogenes* [9]. It also prevented the growth of yeasts and *Enterobacteriaceae*. Similarly, Rey et al. [16] conducted an assay with beef burgers at 600 MPa for 300 s. Under these conditions, native microbiota and *E. coli* O157 were successfully inactivated, extending shelf life during both refrigerated and frozen storage. Three studies have demonstrated the impact of HHP on the physicochemical properties and sensory characteristics of lamb meat, showing that higher-pressure intensities (200–600 MPa at various temperatures) significantly affect parameters, such as pH, color, lipid oxidation, and texture [17]. However, Timón et al. [18] demonstrated that HHP treatments at 600 MPa effectively improved the safety of chicken burgers. Although color changes were observed after processing, these were not perceived at the sensory level after cooking.

Several studies have shown the potential of incorporating vegetable by-products into high-pressure-treated meat products to enhance their stability during storage. For example, Alves et al. [19] added waste from tomato paste to pressurized chicken minced meat, and treatment at 600 MPa increased the lag time for the formation of secondary oxidation substances. Amaro-Blanco et al. [10] added tomato paste to help decrease foodborne growth and increase lipid oxidation stability during storage. Martillanes et al. [20] combined extracts of rice bran with HHP treatment to extend the shelf life of pork burgers free of additives.

Until now, no studies have evaluated the use of cauliflower and broccoli by-products (stems, inflorescences and leaves) as valorized ingredients with bioactive compounds in meat products from lamb. Therefore, this study aimed to analyze the effect of cruciferous by-product ingredients incorporated into lamb burgers, treated with increasing levels of high hydrostatic pressure (400, 500, 600 MPa for 60 s), to extend their shelf life during refrigerated storage.

## 2. Materials and Methods

### 2.1. Material

#### 2.1.1. Manufacture Process of *Brassica* By-Product Ingredients

The *Brassica* by-products consisted of a mixture of stems, inflorescences, and leaves in the following proportions: for broccoli by-products, stems accounted for 45.9% (*w*/*w*), inflorescences 37.3% (*w*/*w*), and leaves 16.8% (*w*/*w*); for cauliflower by-products, stems made up 42.7% (*w*/*w*), inflorescences 48.4% (*w*/*w*), and leaves 8.9% (*w*/*w*). In previous studies (unpublished results), the by-products from *Brassica* were valorized, and ingredients were obtained through the following process: thoroughly washed, trimmed to remove impurities, blanched in boiling water for varying durations (60, 90, 120, and 180 s). After blanching, the activity of the polyphenol oxidase (PPO) enzyme was analyzed in triplicate (n = 3). The optimal blanching time was determined to be 60 s, which reduced PPO activity by 90%. This optimization ensured effective blanching conditions while preserving the quality of the by-products. Subsequently, a puree was processed using a high-shear blender (Thermomix TM5, Wuppertal, Germany) at 100 °C for 1 min. The puree was then evaluated for its bioactive compound content, antioxidant activity, and microbiological safety. These optimized results informed us of the preparation conditions used in this study. The purée (approximately 200 g) was then vacuum-packed in plastic bags (polyamide polyethylene 20/100, with an oxygen permeability of 50 cm^3^ m⁻^2^, 24 h⁻^1^, at 0% relative humidity, 120 µm thickness, Eurobag, Málaga, Spain). Finally, a high-pressure treatment (600 MPa for 300 s at 15 °C) was applied to *Brassica oleracea* var. *botrytis* and *var. italica* to ensure microbiologically safe ingredients. This HHP processing resulted in significant reductions in microbial counts, with mesophilic (control: 6 vs. HHP: <1 log CFU g⁻^1^), *Enterobacteriaceae* (control: 3.6 vs. HHP: <1.7 log CFU g⁻^1^), and molds and yeasts (control: 5.5 vs. HHP: <1 log CFU g⁻^1^) being notably reduced.

#### 2.1.2. Manufacture of Burgers and Inclusion of Brassica By-Product Ingredients into Lamb Burgers

The burgers (with approximately 100 g) were prepared according to a traditional recipe: meat was mixed in a ratio of 40% flank to 60% leg meat, along with 1.3 g 100 g⁻^1^ of salt, 0.15 g 100 g⁻^1^ garlic powder, 0.15 g 100 g⁻^1^ dehydrated onions, and 0.05 g 100 g⁻^1^ black pepper. Lamb meat was provided by a local market. This formulation served as the control lamb burger. Burgers with the by-product ingredients (from broccoli and cauliflower) were prepared using the same base proportions of salt and spices.

**Figure 1:**
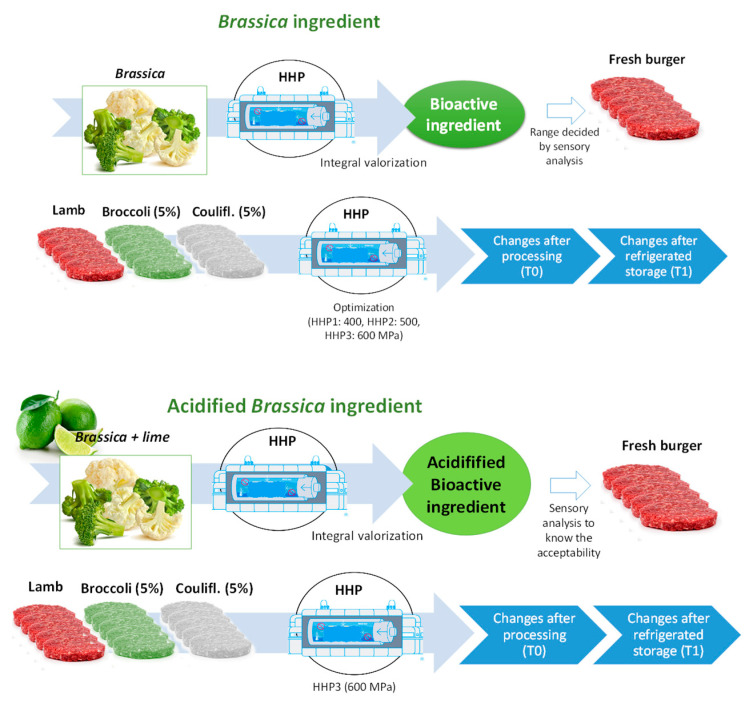
Scheme of the experimental design.

A scheme of the valorization process of the Brassica by-products and the addition to lamb burgers is shown to clarify the experimental design in this study (Figure 1).

The *Brassica* by-product ingredients were incorporated into lamb burgers at the level decided by a sensory analysis. The analysis was carried out following the procedure detailed in Section 2.2.6. For the sensory analysis, an experiment was conducted with four batches containing different levels of the ingredients (less than 20% *w*/*w*). Five burgers per formulation were prepared, resulting in a total of forty burgers studied. Approximately 5 kg of lamb meat (sourced from a local market) was used. The level of 5% ingredient showed high acceptability among panelists, and this proportion was fixed for the subsequent experiments.

#### 2.1.3. Optimization of HHP Conditions on Burgers with *Brassica* By-Product Ingredients

An experiment to optimize the HHP conditions on burgers with *Brassica* ingredients was carried out. Thus, 15 kg of minced lamb meat was utilized for the preparation of burgers, which was mixed with the by-product ingredients at a level of 5% (*w*/*w*), according to results of the previous experiment, and they were treated at different HHP conditions and stored to evaluate changes during storage.

Burgers were individually vacuum-packed in plastic bags (polyamide polyethylene 20/100, with an oxygen permeability of 50 cm^3^ m⁻^2^, 24 h⁻^1^, at 0% relative humidity, 120 µm thickness, Eurobag), and burgers were treated with HHP according to the experimental design and then stored under refrigeration.

Thus, 5 burgers per batch were manufactured to optimize HHP conditions, with a total of 120 burgers produced for the experiment: 3 formulations (lamb, lamb with broccoli, and lamb with cauliflower) × 4 pressure treatments (untreated, 400 MPa, 500 MPa, 600 MPa) × 5 replicates per formulation and pressure treatment × 2 storage times (day 1 and day 14). Additionally, 15 burgers (5 burgers per formulation) were produced for initial characterization (pH, a_w_, proximate composition, and fatty acid profile). Thus, a total of 135 burgers (120 + 15) were manufactured.

A semi-industrial high hydrostatic pressure unit (55 L capacity, Hiperbaric Wave 6000/55; Burgos, Spain) was used to pressurize the vacuum-packed burgers and ingredients. Treated and control burgers were stored in the dark at 6 ± 1 °C for 14 days. Three treatments were applied: 400 MPa for 60 s (HHP1), 500 MPa for 60 s (HHP2), and 600 MPa for 60 s (HHP3), with untreated burgers serving as the control. The initial water temperature inside the pressure vessel was 15 °C. Burgers were analyzed on day 1 (the day after production) and after 14 days of refrigerated storage (6 °C) in darkness.

#### 2.1.4. Manufacture of Acidified *Brassica* By-Product Ingredients, Inclusion in Lamb Burgers and Effect of HHP Treatments

Due to the microbial stability of burgers with *Brassica* by-product ingredients being minimal, an experiment was carried out with new acidified ingredients to improve the microbial stability of burgers. Acidified ingredients from *Brassica* by-products were also prepared to evaluate the effect on fresh burgers’ stability. The acidified ingredient was manufactured following the same process mentioned before, but prior to the packaging and HHP, juice of lime (*Citrus aurantifolia*) was added to lower the initial pH of the broccoli and cauliflower. The initial pH was 6.61 ± 0.06 (broccoli) and 6.56 ± 0.05 (cauliflower), and it was reduced to reach a pH of 4.5 and 5.5, respectively.

The inclusion of these acidified ingredients in burgers was evaluated by sensory analysis to detect if panelists perceived sourness. Five burgers with each acidified ingredient were prepared, cooked and evaluated in a sensory analysis (following the procedure in Section 2.2.6) in a previous and different experiment. Hence, burgers with non-acidified broccoli and non-acidified cauliflower and burgers with the acidified ingredients (5.5 broccoli, 5.5 cauliflower/4.5 broccoli and 4.5 cauliflower) were evaluated and compared. A total of 35 burgers (5 replicates × 7 ingredients) were manufactured and utilized for sensory analysis. For the sensory analysis, 5 kg of minced lamb meat was utilized. No significant differences (*p* ≥ 0.05) were found for all parameters evaluated (appearance, odor, taste, texture, and presence of unpleasant tastes). A pH 4.5 ± 0.01 in the ingredient was selected to ensure the manufacture and stability during the storage of lamb burgers, since the highest acidity of ingredients could provide more safety to burgers.

Once again, the effect of the selected acidified ingredients was evaluated; lamb burgers with the ingredients with pH 4.5 (at 5% *w*/*w*) were prepared following the same procedure as previously described. All burgers were individually vacuum packaged in plastic bags (the same composition as before). Non-treated burgers were compared against HHP burgers, and all of them were stored in refrigeration conditions (at 6 °C) for 14 days. The most intense HHP conditions (HHP3, 600 MPa for 60 s) were chosen to ensure a long shelf life for the burgers (based on microbial inactivation). A total of 60 burgers were prepared for this new experiment: 5 (replicates per batch) × 3 formulations (lamb, acid-broccoli, and acid-cauliflower) × 2 (high-pressure treatments: non-treated, 600 MPa) × 2 (times of storage: day 1 and 14). Here, 7 kg of minced lamb meat (from the same local market) was utilized.

On the other hand, the experiment was replicated twice for the sensory analysis to evaluate the effect of the acidified ingredient (pH 4.5) and HHP. Only samples from day 1 were cooked and tasted (30 burgers: 5 × 3 × 2). Two sessions, each with 30 burgers, were conducted (30 × 2). For this sensory analysis, approximately 8 kg of lamb meat was used to prepare the burgers.

### 2.2. Methods

#### 2.2.1. Proximate Analysis of *Brassica* Ingredients

The moisture, fat, and protein (%) were determined by drying the samples at 104 °C until constant weight, gravimetrically, through extraction with chloroform/methanol (2:1) and using the Kjeldahl method, respectively [21]. Fiber contents of broccoli and cauliflower were assessed through the modified Southgate method [22]. The carbohydrate (%) was calculated by difference. pH was obtained with a pH meter Crison pH 25 + (Crison, Barcelona, Spain), and the water activity (aw) measurement was carried out at 25 °C using a Novasina Labmaster-aw meter (Novasina AG, Lachen, Switzerland), which gives temperature-controlled measurements. Total content of phenolic compounds was measured by the Folin-Ciocalteu reagent-based colorimetric assay [23], and the antioxidant capacity of the extract samples was determined by the ABTS^•+^ method [24].

#### 2.2.2. Proximate Analysis of Burgers and Fatty Acid Profile

The moisture, fat, and protein (%) as well as the pH and water activity (aw) of burgers were evaluated following the same methodology as in the *Brassica* by-products (Section 2.2.1). The fatty acid profile was determined by obtaining the fatty acid methyl esters (FAMEs) using KOH in methanol and analyzing them using Agilent Technologies 6890 GC equipped with a flame ionization detector and an Agilent DB-23 (60 m × 0.25 mm ID × 0.25 μm) column. The injector and detector were maintained at 230 °C, and the oven temperature rose from 185 °C to 220 °C (5 °C min^−1^), with nitrogen as a carrier gas (1.2 mL min^−1^). FAME identification was based on the retention times. Quantification was performed by using tridecanoic acid as internal standard and the response factors determined after injecting solutions (Supelco 37 component FAME mix standard, Sigma Aldrich, St. Louis, MO, USA). Results were expressed as required by the current regulation (Orden PREE/3844/2004).

#### 2.2.3. Microbiological Analyses

Ten grams of burgers was taken aseptically and homogenized with 90 mL of peptone water (Merck, 1.07043, San José, CA, USA) in a laboratory blender (Stomacher^®^ 400 Circulator, West Sussex, UK), and serial decimal dilutions were performed in sterile peptone water and poured or spread onto total count and selective agar plates for *Mesophilic*, *Psychrophilic*, *Molds* and *Yeasts*, *Coliforms, Staphylococcus aureus*, *Clostridium*. The detection limit was 10 CFU g^−1^, except for *S. aureus* (100 CFU g^−1^). The absence in 25 g of sample was evaluated for *Salmonella* sp. and *Listeria monocytogenes* [25].

#### 2.2.4. Instrumental Color

The CIELAB instrumental color measures were acquired using a Minolta CM-5 spectrophotometer (Minolta Camera, Osaka, Japan), with an illuminant/angle of D65/10^0^ and a measuring area of 30 mm. Each burger was analyzed two times, one on each side.

#### 2.2.5. Oxidative Status

Lipid oxidation was assessed using the Thiobarbituric Acid Reactive Substances (TBA-RS) method [26], using a standard curve of 1,1,3,3-Tetraethoxypropane. Protein oxidation was evaluated by measuring the carbonyl groups formed during incubation with 2,4-dinitrophenylhydrazine (DNPH) in 2 N HCl [27]. The absorbance measurement of protein concentration was detected at λ-280 nm using a spectrophotometer (evolution 201 UV-Visible, Thermo Scientific, Waltham, MA, USA) with bovine serum (BSA) as standard. Protein oxidation was expressed as nmol carbonyls mg protein^−1^.

#### 2.2.6. Sensory Analysis

For the sensory analysis and affective testing, two independent sessions on separate days (one for each sensory session) were conducted, with five burgers per batch. Only burgers from day 1 were tasted to ensure the safety of the panelists. The aim of the sensory analysis was to assess the effect of acidified broccoli and cauliflower ingredients (pH 4.5) on the sensory quality of cooked burgers. Burgers with acidified ingredients were treated with HHP (600 MPa for 60 s) and compared with untreated burgers. Thus, a total of 30 burgers (5 replicates × 3 ingredients × 2 HHP conditions) were prepared in each experiment and used for sensory analysis.

The cooking conditions were optimized in advance, and the internal temperature of each burger was controlled (72 °C). Each burger was cooked on a contact grill (Lacor, 43 × 35 × 19 cm; 1400 W) at maximum temperature (220 °C) for 120 s. After cooking, each burger was cut into four pieces and wrapped in aluminum foil. A random identification number was assigned to each batch. Mineral water and bread were provided to each panelist. Each panelist tasted one piece from each batch. Twenty untrained panelists between the ages of 20 and 65 participated in the sensory analysis. The following parameters were evaluated using an unstructured scale (from “I dislike it very much” to “I like it very much”): general appearance, odor, taste, texture, and overall acceptability. Additionally, the presence or absence of undesirable flavor was assessed (absence/presence).

### 2.3. Statistical Analysis

Results are expressed as mean and standard deviation. A three-way ANOVA was conducted to evaluate the individual and interaction effects of burger formulation, treatment, and storage on the results. Unidirectional analysis of variance (ANOVA) was used to analyze differences due to different treatments using the SPSS 21.0 statistical program (SPSS Inc., Chicago, IL, USA). In addition, the Student test was applied on each day of storage, day 1 and day 14, respectively. When the ANOVA showed a significant effect (*p* ≤ 0.05), the Tukey test was performed to compare, in pairs, the sample groups.

## 3. Results and Discussion

### 3.1. Characterization of Ingredients from Broccoli and Cauliflower By-Products

The proximate composition, pH, and antioxidant activity of ingredients are shown in Table 1. The results agree with those reported in the literature [28,29]. Broccoli and cauliflower had a moisture content higher than 90. Water activity (a_w_) was higher in cauliflower than in broccoli, as well as the moisture content. The pH reached values of 6.5 in cauliflower and 6.0 in broccoli. pH values were near neutral, which could present potential issues for future food applications due to the increased likelihood of microbial growth. In fact, these by-products were added to the burgers at low levels (<5%), showing significant statistical differences in burger pH (Section 3.2). This could compromise the hygienic quality of the final products, which will be discussed in Section 3.4. These ingredients also contained a high fiber content (2.1 and 2.8 (%)), which is beneficial for meat product manufacturing as it improves the technological properties [30]. The protein content of broccoli and cauliflower also agrees with the results from Madhu et al. [29] and Reis et al. [28]. The fiber and fat contents reported by Reis et al. [28] for cooked inflorescences of broccoli (*Brassica oleracea* var. *Avenger*) and cauliflower (*Brassica oleracea* var. *Alphina F1*) were higher than the values observed in our study. In general, the biochemical composition varies according to the species and part of the *Brassica* plant analysed, the cultivar, fertilization, environment [6], state of maturity, and the type of gather [31]. The by-product ingredients in this study were mostly manufactured with a mixture of florets, stems, and leaves, not only with the florets (which is the most consumed part of the vegetable), which may explain some compositional differences compared with previous studies.

The phenolic content in ingredients was lower than that reported for fresh broccoli (34–337 mg 100 g^−1^) and cauliflower (28–274 mg 100 g^−1^) by Reis et al. [6,28]. This could be caused by the transformation process of the by-product (thermal blanching, blending, HHP), since some reductions in bioactive compounds are expected after processing compared to the fresh vegetable. The antioxidant capacity of the cauliflower ingredient was significantly higher than that of broccoli (*p* < 0.001), despite its phenolic content being the lowest (*p* < 0.001) (Table 1). This highlights that antioxidant activity is not solely determined by phenolic content but can also be influenced by other compounds, such as vitamin C and lipid-soluble antioxidants (e.g., carotenoids and vitamin E), which were not quantified in this study. Nonetheless, the antioxidant activity of both broccoli and cauliflower falls within the range reported in previous studies [32,33].

### 3.2. Initial Proximate Composition of Burgers with and Without Broccoli and Cauliflower Ingredients

Table 2 shows the pH, a_w_ values, moisture, protein and fat content as well as fatty acid profile in processed standard burgers (without broccoli or cauliflower ingredients) and burgers with added broccoli or cauliflower ingredients. pH values were slightly higher than those in other studies with aqueous extracts from pomegranate, olive, red grape and tomato pomaces [34,35] or guarana (*Paullinia cupana*) seed and pitanga (*Eugenia uniflora* L.) leaf extracts [36]. The pH values for the lamb, broccoli, and cauliflower burgers were 5.71, 5.73, and 5.75, respectively, showing a statistically significant difference (*p* = 0.020). Although these differences are statistically significant, they are quite low and likely do not have a substantial practical impact on the technological or microbiological quality of the burgers. In this sense, Martínez-Zamora et al. [37] reported that a pH higher than 6 for lamb burgers may promote microbial growth, which was not reached in any of the current treatments.

The water activity (a_w_) values did not differ significantly among the three types of burgers, with values remaining close to 0.93 (*p* > 0.05). This indicates that the addition of broccoli and cauliflower by-products does not significantly increase the water activity, which would otherwise enhance the risk of microbial growth and reduce the shelf-life stability of burgers in the present experiment [34,35,36,38].

In Table 2, the moisture content is significantly higher in burgers with broccoli (67.21 ± 0.40%) and cauliflower (66.19 ± 0.45%) compared to lamb burgers (62.85 ± 0.59%) (*p* = 0.000). This increase in moisture can be attributed to the high water content in broccoli and cauliflower by-products (Table 1) and may significantly impact positively on sensory parameters, particularly texture attributes, increasing the juiciness, as observed by Fernandes et al. [39]. The protein content was slightly lower in burgers with added broccoli and cauliflower, but the differences were not statistically significant (*p* = 0.195). The fat content showed no significant differences among the three types of burgers (*p* = 0.397), indicating that the inclusion of vegetable by-products does not significantly affect the overall fat content. The relatively stable protein and fat contents suggest that the proximate composition of the burgers is maintained despite the addition of vegetable by-products, which could be caused by the low level of *Brassica* by-products added to the hamburger patties.

The proximate composition (Table 2) and fatty acids profile (Table S1) of lamb burgers were like those reported by previous studies, although the fat content was slightly lower than similar burgers in earlier studies [37,38] and higher than those obtained by Carvalho et al. [35,40].

### 3.3. Color and Oxidative Changes in Lamb Burgers Treated by High Hydrostatic Pressure

Lamb burgers with broccoli and cauliflower by-product ingredients were treated at different HHP conditions (400, 500, 600 MPa for 60 s; HHP1, HHP2, and HHP3, respectively), and the changes after processing (T0) and after 14 days of refrigerated storage (T1) were evaluated. In general, the color parameters of burgers were affected by the added *Brassica* ingredients and by HHP, as well as their interaction (*p* = 0.000) (Table 3), whereas the time of storage had a more limited effect. The addition of broccoli or cauliflower into burgers promoted a decrease in redness with independence of refrigerated storage; that is, burgers were less red one day after manufacture (T0) as well as after 14 days of storage (T1 samples) (*p* ≤ 0.05) (Table 4). This behaviour can be also inferred from Table 3, where there was no interaction between these factors (ingredient × storage; F1 × F3; *p* ≥ 0.05). The lower a* value in the broccoli and cauliflower burgers might be due to the decrease in the myoglobin concentration caused by the presence of plant-based ingredients in the formulation. These results are consistent with findings from other studies, in which the addition of vegetable ingredients to meat patties resulted in changes in redness [36,40,41,42].

CIE a* and b* values significantly changed with days of storage (*p* ≤ 0.05), regardless of the added ingredient, as has been mentioned (Table 4). The loss of intensity of red colour (a*) of fresh lamb meat during storage has been previously observed [17,34,43,44]. It is due to the oxidation of oxymyoglobin to metmyoglobin, the pigment responsible for the brown discoloration of meat [39].

Regarding the effect of HHP on the colour of lamb burgers, it was statistically significant on every color parameter (*p* ≤ 0.001) (Table 3). It is known that HHP induces significant alterations in the color of fresh meat (a* value), although changes are not generally perceived after cooking, which poses a challenge for commercialization due to the changed typical color, an essential parameter for consumers. This alteration complicates the marketing of these products. Consequently, one of the primary objectives in formulating fresh meat products with vegetable by-products was to mitigate the discoloration promoted by HHP, leveraging the potential antioxidant effect of active compounds present in these by-products. However, a* values decreased in HHP-treated samples compared to non-treated ones (*p* < 0.05), while b* values showed a slight decrease in non-treated lamb burgers, those containing cauliflower, or lamb burgers with broccoli + HHP2. This suggests that HHP treatment can influence the redness and yellowness of meat patties both one day after manufacturing (T0) and after 14 days of storage (T1) (Table 4). Moreover, the addition of vegetable ingredients was not effective in reducing discoloration. In fact, it had the opposite effect. Amaro-Blanco et al. [10] did not observe a lower discoloration in burgers with tomato paste after HHP (600 MPa, 300 s) comparted to burgers without that vegetable ingredient. However, tomato paste composition (pigments, antioxidants, pH, etc.) and colour are very different to *Brassica* ingredients, and their effect on meat color was very different. In addition, the increase in pressure from 400 MPa to 500 and 600 MPa (HHP1, HHP2 and HHP3) promoted a significant decrease in a* values. The observed decrease in a* values due to HHP can be attributed to the oxidation of ferrous myoglobin to ferric metmyoglobin, leading to brown discoloration [34]. Coincidentally, the literature describes reductions in the redness of meat at pressures between 200 and 500 MPa [10] and 200 and 600 MPa in loin cuts (Ma et al. [44]), and it is well established that HHP has a notable effect on the structural characteristics of proteins in meat, which, in turn, affects the color of the meat [45].

HHP promoted an increase in the L* values of lamb burgers, no matter the treatment (lamb, broccoli or cauliflower) or time of storage (T0 or T1) (Table 4). Coincidentally, Jung et al. [46] reported an increase in L* values in beef *M. biceps femoris* samples after HHP (350–600 MPa). Other authors have suggested that this “whitening/brightening” effect of pressure could be attributed to globin denaturation, the displacement of heme iron or release, and ferrous atom oxidation [8]. On the other hand, pressure can induce changes in protein structures, such as unfolding [45], which can affect how proteins interact with light. This results in changes to the meat lightness, making it appear lighter in color. This effect can be particularly evident in products such as ground meat or processed meats, where the proteins are more exposed to pressure and undergo significant changes [43].

Regarding lipid stability, TBA-RS values were significantly affected by ingredients, HHP and refrigerated storage (Table 3), along with their interactions. Some authors have observed that pressure levels between 300 and 600 MPa are critical for inducing lipid oxidation in fresh pork, beef, and poultry meat [14,45], which can lead to rancidity, off-flavors, and decreased shelf life, ultimately affecting the quality and consumer acceptance of the meat products. In this experiment, the addition of broccoli and cauliflower by-products significantly increased the lipid oxidative stability of both non-treated and treated HHP lamb burgers (Table 4). The addition of vegetable ingredients in general also promoted a higher protein oxidation stability in HHP samples. At day 14, protein oxidation tended to increase in most burgers, although changes were not significant in all cases, and the overall values of protein oxidation remained lower in burgers with these ingredients. Both the lipid and protein oxidation results can be attributed to the presence of antioxidant compounds in the added by-products, as previously reported in cauliflower and broccoli [5,6,7].

### 3.4. Microbial Counts in Lamb Burgers Treated by High Hydrostatic Pressure

A three-way ANOVA (Table 3) showed the global effect of the studied factors (type of ingredient, HHP treatment and time of storage) on the initial counts (log CFU g^−1^ lamb burger with or without *Brassica*) of mesophilic, psychrophilic, molds and yeasts, and coliforms (*p* ≤ 0.001). *S. aureus* and *Clostridium perfringens* were affected by the type of ingredient and HHP, while the refrigerated storage had no effect. Table 5 displays the microbial counts for each of the treatments of burgers. High microbial loads were found in non-treated lamb burgers, although the absence of *Salmonella* sp. and *Listeria monocytogenes* in 25 g was confirmed in all samples. The counts of non-treated burgers were in accordance with the recommendations of Commission Regulation (EC) No 2073/2005 [47]. In non-treated lamb burgers at T0, mesophilic counts led to a significant increase due to the addition of broccoli or cauliflower, while coliforms and *Clostridium* counts increased due to the addition of cauliflower. In this sense, manufacture conditions, particularly regarding the microbial quality of by-product ingredients, and optimization of the physical–chemical composition, which could affect microorganisms’ development, should be carefully improved for forthcoming experiments.

There was an increase in mesophilic, psychrophilic, coliforms, and mold and yeast count after 14 days of refrigerated storage, across all treatments (*p* ≤ 0.05) (Table 5). Regarding interactions with the added ingredients, counts were even higher for burgers containing cauliflower, except molds and yeasts, after 14 days under refrigeration (Table 3 and Table 5).

HHP treatment significantly reduced microbial counts, even at the lowest pressure level of 400 MPa (Table 5) at T0. Mesophilic counts (log CFU g⁻^1^) decreased in lamb, broccoli and cauliflower burgers after HHP1, HHP2 and HHP3. Similarly, psychrophilic counts were reduced by HHP, with *Brassica*-containing burgers showing counts lower than 2 log CFU g⁻^1^. The mold and yeast count decreased from 2.8 to 4.8 for non-treated burgers with or without *Brassica* to approximately 1.0 by HHP3 (600 MPa for 60 s). Meanwhile, in non-treated cauliflower burgers, the coliforms count dropped from 1.0 to undetectable levels in HHP3-treated samples. The interactions with added ingredients were statistically significant, except for *S. aureus*. Considering the high counts of lamb burgers (with or without ingredients), the application of the most intense HHP conditions (HHP3) is recommended to reach the maximum inactivation of microorganisms and to ensure the safety of the treated products during the refrigerated storage. The effect of HHP to increase microbial safety in burgers aligns with the findings of Amaro-Blanco et al. [25], who demonstrated the effectiveness of HHP treatment (600 MPa for 300 s) in inactivating spoilage and pathogenic microorganisms in pork burgers with tomato paste. Under those conditions (a longer holding time than HHP3, and an acidified burger due to the addition of tomato paste), HHP effectively reduced mesophilic, psychrophilic, *S. aureus*, *E. coli*, and *C. perfringens* counts.

When combined with natural antimicrobial substances or ingredients, HHP could be an effective strategy for inactivating *C. perfringens* spores and other foodborne bacteria in meat products, demonstrating synergistic effects. These additional hurdles could prevent the growth of sublethal injured cells after HHP treatment [16,45]. Incorporating vegetable by-products into lamb burgers could further extend product shelf life, as suggested by Andres et al. [33]. This could be a strategy to increase the shelf life of treated HHP burgers. However, in our case, the initial microbial counts of burgers were increased when broccoli and cauliflower ingredients were added, probably caused by the composition of these by-products (high pH and moisture content), which favour microbial development. Therefore, for burgers enriched with these ingredients, the application of the most intense HHP treatment, like HHP3, would be necessary to ensure the safety of the burger. Moreover, this type of vegetable by-product has a low phenolic content, which is a key factor in antimicrobial activity.

Additionally, pH levels above 6 in lamb burgers may encourage microbial proliferation [37]. pH is a critical factor influencing microbial growth during refrigerated storage and especially in treated HHP products, as confirmed by Bolumar et al. [45]. A combination of pH control, vacuum packaging, and refrigeration could synergistically prevent the recovery of sublethal injured cells after HHP, thereby extending the shelf life [45]. Various studies have demonstrated a significant inactivation of *L. monocytogenes*, *Salmonella* sp., and *S. aureus* with pressure levels up to 400 MPa, considering factors like pH and water activity to inhibit the growth and survival of pressure-damaged microorganisms [14]. In our study, HHP treatments maintained the safety in lamb burgers with *Brassica* by-products but with a short shelf life (less than 14 days), since at T1, most burgers were out of the recommendable microorganisms’ counts limits (Commission Regulation (EC) No 2073/2005) [46], so that changes in the composition of *Brassica* by-product ingredients would be required to enhance the preservation of lamb burgers. Despite improvements in the *Brassica* ingredients, microbial counts in non-HHP-treated burgers exceeded regulatory limits after 14 days of storage. Therefore, HHP (under the conditions tested in this study or even more intense conditions) would be necessary to ensure the safety of these enriched burgers.

### 3.5. Selection of the Optimum Acidified Ingredients from Brassica By-Products and Manufacture of High-Pressure-Treated Lamb Burgers

It would be reasonable to hypothesize that supplementary hurdles, such as reducing the pH of burgers through the addition of acidified *Brassica* by-product ingredients before applying HHP, would serve as a valid and innovative method to ensure the safety of processed meat products. To enhance the logical connection, the impact of acidified *Brassica* by-products on TBA-RS values, pH, and microbial counts is discussed separately, followed by the evaluation of HHP treatment.

The improved acidified ingredients from *Brassica* by-products presented the following composition: broccoli (pH: 4.5 ± 0.01; a_w_: 0.942 ± 0.003; moisture 89.30 ± 0.10 (%)) and cauliflower (pH: 4.5 ± 0.01; a_w_: 0.944 ± 0.001; moisture 92.10 ± 0.10 (%)). These optimized ingredients were utilized for manufacturing lamb burgers (5% *w*/*w*), which were later processed at HHP3 conditions (600 MPa, 60 s) and stored for 14 days at 6 °C.

The results for pH, aw, instrumental color, lipid oxidation (TBA-RS values), protein oxidation (carbonyl content), microbial counts, and sensory analysis of the manufactured lamb burgers are shown in Table 6. The pH values ranged from 5.8 to 6.0 and were not significantly affected by the low levels of added acidified Brassica ingredients (*p* ≥ 0.05). According to these results, it seems that the low levels of addition of the *Brassica* ingredients were not sufficient to significantly reduce the pH of the burgers. However, the inclusion of the acidified ingredients did not increase the pH. The HHP treatment was observed to increase the pH of the samples, resulting in higher pH levels compared to untreated samples (*p* < 0.05). This agrees with the findings of McArdle et al. [17], who reported an increase in pH values for lamb *M. pectoralis profundus* samples subjected to high pressure levels of 300 and 400 MPa. With reference to the values of a_w_, a_w_ values remained unchanged (*p* > 0.05) by either the HHP treatment or the addition of *Brassica* ingredients, aligning with earlier results in this study (Table 2).

Regarding instrumental color, the acidified ingredients did not significantly affect L* and a* values in T0 burgers. However, after 14 days of storage (T1), the lightness was increased, while the redness was decreased in all burgers. In general, these trends were similar to those found in the experiment with the non-acidified ingredients. However, in this case, at T1, broccoli-added burgers presented lower lightness and higher redness than lamb or cauliflower burgers (Table 6). The addition of the acidified broccoli ingredient appears to have stabilized the red color and lightness in non-treated burgers during storage; however, this effect is unexpected as it was not observed in the non-acidified by-product. On the other hand, as in the previous experiment of this study, HHP treatment at 600 MPa resulted in slight color changes, with increased lightness (L*) and reduced redness (lower a* values) across all samples (*p* < 005).

The acidified ingredients did not significantly affect TBA-RS values. Unlike the non-acidified Brassica by-products, the acidified versions did not exhibit antioxidant effects. Concerning the effect of HHP treatment, initial TBA-RS values were slightly higher in HHP-treated samples than in non-treated ones, aligning with previous findings that report that lipid oxidation tends to increase with higher HHP pressures applied to lamb cuts [43,44]. Regarding storage time, TBA-RS values significantly increased (*p* < 0.05) in lamb, broccoli, and cauliflower burgers from 0.4 mg MDA kg^−1^ to 0.9, 1.0, and 1.0 mg MDA kg^−1^, respectively, after 14 days. In treated HHP samples, changes during storage were not significant (*p* > 0.05), and the values ranged from 0.5 to 0.6 mg MDA kg^−1^ at T0 to 0.7 mg MDA kg^−1^ at T1. So, treated HHP burgers showed more stability in lipid oxidation during storage than non-treated burgers. On the contrary, Timón et al. [18] reported that treated HHP chicken burgers showed a more intense increase in TBA-RS than non-treated samples during storage. Differences in the effect of HHP on burgers during storage might be caused by the different composition of meat spices (chicken vs. lamb) and their oxidative stability during processing and storage (fatty acids profile, antioxidant content, etc.).

Protein oxidation, measured based on the carbonyl content, was significantly affected by the addition of ingredients, lamb and broccoli burgers (either non-treated HHP or treated HHP) showing the lowest values for carbonyls (*p* < 0.05), and the highest for cauliflower burgers (Table 6). Therefore, the acidification of these vegetable ingredients—particularly cauliflower—may lead to a lower protein stability, which could negatively impact burger quality. However, this does not appear to have been the case in this experiment, judging from the results of the sensory analysis. The carbonyl values align with those previously found by Amaro-Blanco et al. [10], who observed similar levels of protein oxidation (2.6–3.4 Nmol carbonyls mg protein^−1^) in pork burgers processed with tomato paste under high pressure (600 MPa, 300 s). Protein stability decreased with storage in non-treated and treated HHP samples, but a less intense increase in carbonyls can be observed in the case of treated HHP samples. These results are consistent with those of Timón et al. [18], who did not observe increases in protein oxidation at pressure intensities between 400 and 600 MPa, applied to chicken burgers. The high levels of protein oxidation in non-treated samples at T1 were unexpected and may be associated with greater microbial growth in these samples compared to the pressurized ones. In this regard, Wang et al. [48] identified a significant relationship between microbial spoilage and lipid and protein oxidation in meat during storage. These authors hypothesized that microbial growth could lead to the production of enzymes that degrade lipids and proteins, making tissues more susceptible to oxidative changes.

The high microbial load of *Brassica* by-products, combined with the limited reduction in the initial load by HHP, may necessitate the combination of HHP with other pretreatments, such as the addition of preservatives like ascorbic acid or the application of more intense thermal blanching conditions alongside HHP. Even intense thermal treatments, like sterilization, could be suitable for improving the safety of *Brassica* by-products. However, such treatments often reduce the content of bioactive compounds in vegetable products [13], which is why HHP was chosen as the preferred treatment.

The acidified ingredients (broccoli or cauliflower) did not significantly affect microbial counts in burgers at T0 (Table 6), which is expected, as their addition did not alter the pH. In this case, the use of the vegetable ingredient did not promote microbial growth, unlike in the previous experiment (Table 5), and burgers containing broccoli and cauliflower showed similar counts to the control. Previous studies suggest that using acidified by-products may enhance the microbial safety of these products. For example, the incorporation of agri-food by-products into lamb burgers has been explored from a technological perspective, particularly in relation to microbiological stability [36]. Studies utilizing acid by-products with low water activity (a_w_) in burger formulations have demonstrated improved stability even at low inclusion levels (<3%) [49]. Similarly, Martín-Mateos et al. [50] added a low-pH ingredient derived from grape pomace to pork burgers, which improved their preservation. However, in this case, the pH effect alone was insufficient to extend the shelf life of the burgers, likely due to the high moisture content of the ingredient. Further studies should focus on improving the reduction in moisture content. However, if the reduction is too extensive, it may reduce the effectiveness of HHP in inactivating microorganisms during the valorization process, since the inactivation of microorganisms by pressure is less intense in products with low a_w_ [51].

The effect of HHP treatment on microbial counts was significant (Table 6), with the lowest counts observed in treated HHP burgers at both T0 and T1, despite the initial high counts. HHP treatment significantly reduced the counts of mesophilic, psychrophilic bacteria, molds and yeasts, coliforms, *S. aureus*, and *Clostridium* across all burgers (*p* < 0.05), consistent with previous studies on burgers treated at similar pressure levels [10,18]. Specifically, HHP led to reductions of 3.4–3.6 log in mesophilic counts, and this reduction was maintained throughout storage.

During storage, mesophilic and psychrophilic counts generally increased in this study, while molds, yeasts, and coliform counts tended to decrease (though changes were not significant in all groups). The reduction in molds and yeasts may be attributed to the vacuum packaging of the burgers, as molds are primarily aerobic. However, the significant reduction in coliforms in non-treated burgers during storage contrasts with the increases observed in burgers containing the non-acidified ingredient (Table 5). Differences in the initial counts or coliform strains may account for these variations.

Using multiple preservation hurdles for meat products—such as reducing matrix pH, introducing antimicrobial substances, and applying HHP—can effectively inactivate microorganisms by preventing the recovery of sublethal injured cells, thereby increasing the safety and shelf life of meat products [45]. For example, the application of HHP to pork burgers manufactured with concentrated tomato [10] significantly improved the microbial stability. However, in our study, the acidified *Brassica* ingredients had a high moisture content, which could have led to the fact that their inclusion in burgers did not provide an antimicrobial effect. Therefore, HHP could be a valuable strategy to enhance the safety of lamb burgers enriched with *Brassica*, as the ingredient did not alter the pH of the burgers. In this case, lamb burgers with *Brassica* by-products treated at 600 MPa for 60 s comply with Commission Regulation (EC) No 2073/2005 [47], while other groups did not. Thus, the inclusion of *Brassica* in burgers should be paired with HHP treatment to ensure product safety.

Regarding the sensory analysis results, Table 6 shows no significant differences among treatments (*p* > 0.05), with all cooked lamb burgers receiving high scores for appearance, odor, taste, texture, and overall evaluation. The acidification of by-products proved to be an effective method for enhancing microbial safety. However, when added in high proportions to meat products, it may affect organoleptic properties. In this case, the addition was limited to a low proportion (5%), which was not perceptible at the sensory level. Thus, some previously mentioned undesirable effects, such as color changes and increased oxidation, were not detected by panelists. This suggests that the incorporation of *Brassica* ingredients and the application of HHP did not negatively affect the sensory characteristics of the lamb burgers. These findings are consistent with previous studies, which reported no sensory differences between treated HHP and non-treated burgers after cooking, despite some changes in color or texture parameters in burgers made from other species, such as chicken [18] and pork [10,20,43,44].

## 4. Conclusions

The incorporation of acidified *Brassica* by-products, particularly broccoli and cauliflower, in lamb burger formulations, alongside high hydrostatic pressure (HHP) treatment, offers a promising approach for enhancing the microbial safety and shelf life of lamb meat products. This study demonstrated that while the addition of these vegetable ingredients slightly impacted the physicochemical properties—such as moisture and fatty acid composition—the overall nutritional profile remained stable. However, the high moisture content of *Brassica* by-products, combined with their limited effect on pH reduction, did not exhibit a strong antimicrobial effect independently. Therefore, pairing these by-products with HHP treatment (600 MPa for 60 s) proved essential to achieving significant microbial reductions, complying with Commission Regulation (EC) No 2073/2005.

HHP treatment effectively reduced microbial counts across all measured groups, which was sustained throughout refrigerated storage, highlighting its value as a non-thermal intervention for food safety. Notably, while HHP caused slight color changes due to myoglobin oxidation, these did not negatively impact the sensory attributes. Panelists found no significant differences in appearance, odor, taste, or texture between treated and untreated samples, and acceptance levels remained high. This suggests that HHP can be successfully applied to *Brassica*-enriched lamb burgers without compromising consumer perception.

Finally, the integration of acidified vegetable by-products with HHP treatment in lamb burgers could serve as a synergistic antibacterial strategy and an efficient, eco-friendly approach for extending shelf life and ensuring safety in meat products while preserving sensory quality. Further research may explore the optimization of by-product moisture content and other hurdle technologies, such as pH control, to amplify the antimicrobial effects and optimize product stability.

## Figures and Tables

**Table 1:** Initial characterization of the optimized ingredients from broccoli and cauliflower by-products used in the preparation of lamb burgers.

	Broccoli	Cauliflower	*p*-Value
pH	6.0 ± 0.0	6.5 ± 0.0	0.000
a_w_	0.988 ± 0.003	0.993 ± 0.003	0.026
Brix	10.8 ± 0.3	9.5 ± 0.1	0.000
**Proximate composition** (%)			
Moisture	91.10 ± 0.10	92.75 ± 0.10	0.000
Protein	2.89 ± 0.24	2.01 ± 0.30	0.001
Fat	0.31 ± 0.01	0.34 ± 0.01	0.003
Carbohydrates	2.89 ± 0.31	2.79 ± 0.34	0.631
Fiber	2.80 ± 0.07	2.10 ± 0.09	0.000
Phenolic compounds (mg 100 g^−1^)	15.98 ± 0.64	6.90 ± 0.09	0.000
Antioxidant activity (mM Trolox g^−1^)	0.20 ± 0.12	0.64 ± 0.01	0.000

Student’s *t*-test. Significant at *p* ≤ 0.05; very significant *p* ≤ 0.01; highly significant at *p* ≤ 0.001.

**Table 2:** Initial characterization of lamb burgers made with or no broccoli and cauliflower by-product.

Burgers	Lamb	Broccoli	Cauliflower	*p*-Value
pH	5.71 ± 0.0 b	5.73 ± 0.0 ab	5.75 ± 0.0 a	0.020
a_w_	0.924 ± 0.004	0.929 ± 0.003	0.927 ± 0.003	0.254
**Proximate composition** (%)				
Moisture	62.85 ± 0.59 b	67.21 ± 0.40 a	66.19 ± 0.45 a	0.000
Protein	19.65 ± 0.20	19.26 ± 0.31	19.05 ± 0.09	0.195
Fat	10.12 ± 0.71	9.57 ± 0.92	11.00 ± 0.46	0.397

Means values followed by different letters indicate the existence of significant differences between treatments by the Tukey test. Significant at *p* ≤ 0.05; very significant *p* ≤ 0.01; highly significant at *p* ≤ 0.001.

**Table 3:** Three-way ANOVA. Global effect of the evaluated factors on processed lamb patties: F1 (effect of added ingredient made from broccoli or cauliflower by-products); F2 (effect of high hydrostatic pressure treatment HHP); F3 (effect of refrigerated storage for 14 days).

	Ingredients (F1)	HHP (F2)	Refrigerated Storage (F3)	F1 × F2	F1 × F3	F2 × F3	F1 × F2 × F3
**Color**							
CIE L*	0.187	**0.000**	0.273	0.210	0.079	0.957	0.740
CIE a*	**0.000**	**0.000**	**0.030**	**0.004**	0.851	0.201	0.499
CIE b*	**0.000**	**0.000**	0.530	**0.000**	0.179	0.112	**0.002**
**Oxidation**							
TBA-RS	**0.000**	**0.000**	**0.000**	**0.000**	**0.000**	**0.000**	**0.003**
Protein oxidation	**0.000**	**0.000**	**0.000**	**0.000**	0.412	**0.000**	**0.000**
**Microbiology**							
*Mesophilic*	**0.000**	**0.000**	**0.000**	**0.002**	**0.000**	**0.000**	**0.001**
*Psychrophilic*	**0.000**	**0.000**	**0.000**	**0.000**	**0.000**	**0.000**	**0.000**
*Molds and yeasts*	**0.048**	**0.000**	**0.000**	**0.001**	**0.086**	**0.000**	**0.000**
*Coliforms*	**0.000**	**0.000**	**0.000**	**0.000**	**0.000**	**0.000**	**0.000**
*S. aureus*	**0.003**	**0.000**	0.944	0.659	**0.035**	**0.024**	0.928
*Clostridium*	**0.000**	**0.009**	0.173	**0.000**	0.889	0.202	**0.008**

F = Factor.

**Table 4:** Instrumental color and lipid and protein oxidation of lamb burger with *Brassica* by-products.

	Non-Treated			HHP1			HHP2			HHP3		
	Lamb	Broccoli	Cauliflower	Lamb	Broccoli	Cauliflower	Lamb	Broccoli	Cauliflower	Lamb	Broccoli	Cauliflower
CIE L*												
T0	51.8 ± 1.8 c	50.4 ± 3.6 c	53.5 ± 2.7 bc	56.6 ± 3.1 ab	57.1 ± 0.6 ab	55.9 ± 2.0 ab	58.4 ± 2.1 a	57.4 ± 1.6 ab	60.4 ± 0.7 a	59.4 ± 2.7 a	59.6 ± 2.2 a	59.8 ± 1.2 a
T1	51.7 ± 2.2 d	53.5 ± 2.6 cd	52.7 ± 1.1 d	57.3 ± 2.7 ab	57.4 ± 2.6 ab	56.3 ± 1.6 bc	57.5 ± 1.9 ab	59.2 ± 1.7 ab	59.9 ± 1.2 ab	58.8 ± 1.7 ab	61.0 ± 1.8 a	60.0 ± 1.6 ab
	ns	ns	ns	ns	ns	ns	ns	ns	ns	ns	ns	ns
	CIE a*											
T0	7.4 ± 0.9 a	6.0 ± 0.9 b	5.8 ± 1.0 b	4.6 ± 0.8 b	3.4 ± 0.8 c	4.9 ± 0.6 b	3.3 ± 0.6 c	2.3 ± 0.7 c	2.9 ± 0.3 c	2.3 ± 0.8 c	2.0 ± 0.6 c	2.3 ± 0.4 c
T1	7.3 ± 0.3 a	6.2 ± 0.6 a	6.3 ± 0.4 a	4.8 ± 1.5 b	2.6 ± 0.9 cd	3.9 ± 0.9 bc	2.4 ± 1.1 cd	2.1 ± 0.7 d	2.5 ± 0.7 cd	1.9 ± 0.7 d	1.9 ± 0.2 d	1.6 ± 1.0 d
	ns	ns	ns	ns	ns	ns	ns	ns	ns	ns	ns	ns
	CIE b*											
T0	9.5 ± 0.3 d	11.3 ± 1.3 abc	11.4 ± 0.6 abc	10.2 ± 0.3 cd	11.6 ± 0.6 ab	12.2 ± 0.8 a	10.4 ± 0.4 bcd	12.2 ± 0.7 a	11.3 ± 0.6 abc	10.8 ± 0.6 abcd	11.3 ± 0.6 abc	10.2 ± 0.8 bcd
T1	8.9 ± 0.5 e	11.8 ± 0.9 ab	9.9 ± 0.5 de	10.7 ± 0.4 bcd	12.1 ± 1.1 a	11.5 ± 0.5 abc	10.7 ± 0.4 bcd	11.0 ± 0.9 abcd	11.6 ± 0.5 abc	11.1 ± 0.4 abcd	11.7 ± 0.7 abc	10.4 ± 0.6 cd
	*	ns	*	ns	ns	ns	ns	*	ns	ns	ns	ns
	*TBA-RS MDA (mg kg^−1^)*											
T0	0.79 ± 0.03 a	0.45 ± 0.03 de	0.72 ± 0.06 ab	0.65 ± 0.07 abc	0.51 ± 0.05 cde	0.57 ± 0.03 bcd	0.76 ± 0.15 a	0.37 ± 0.07 e	0.56 ± 0.14 cd	0.77 ± 0.07 a	0.39 ± 0.06 e	0.58 ± 0.4 bcd
T1	0.96 ± 0.07 d	0.5 ± 0.04 f	0.8 ± 0.03 de	1.24 ± 0.17 c	0.6 ± 0.07 ef	0.89 ± 0.03 d	1.47 ± 0.15 ab	0.60 ± 0.1 ef	1.20 ± 0.08 c	1.66 ± 0.14 a	0.67 ± 0.19 ef	1.28 ± 0.17 bc
	**	ns	*	***	**	***	***	**	***	***	*	***
	*Protein oxidation carbonyls (Nmol mg^−1^ protein)*											
T0	1.23 ± 0.12 d	1.96 ± 0.20 bcd	2.54 ± 0.43 bcd	3.17 ± 0.90 b	1.70 ± 0.58 cd	2.14 ± 0.29 bcd	7.56 ± 1.36 a	2.77 ± 0.72 bc	2.64 ± 0.31 bcd	2.13 ± 0.62 bcd	1.79 ± 1.03 bcd	3.15 ± 0.52 b
T1	3.94 ± 0.89 ab	2.42 ± 0.33 b	3.27 ± 0.64 b	3.63 ± 0.64 ab	2.66 ± 0.35 b	3.73 ± 0.49 ab	3.05 ± 0.4 b	3.14 ± 0.35 b	3.28 ± 0.7 b	5.05 ± 1.43 a	2.23 ± 0.25 b	3.54 ± 1.61 ab
	**	*	ns	ns	*	***	***	ns	ns	**	ns	ns

T0: 1 day of storage T1: 14 days of storage. Broccoli: lamb burgers wit 5% of broccoli by-product. Cauliflower: lamb burgers wit 5% of cauliflower by-product. HHP1: 400 MPa 60 s; HHP2: 500 MPa 60 s; HHP3: 600 MPa 60 s. Means values followed by different letters indicate the existence of significant differences between treatments by the Tukey test (*p* ≤ 0.05). ns indicates no difference; * Significance at *p* ≤ 0.05; ** Significance at *p* ≤ 0.01; *** Significance at *p* ≤ 0.001.

**Table 5:** Microbiological counts (log CFU g^−1^) of lamb burgers with *Brassica* by-products.

	Non-Treated			HHP1			HHP2			HHP3		
	Lamb	Broccoli	Cauliflower	Lamb	Broccoli	Cauliflower	Lamb	Broccoli	Cauliflower	Lamb	Broccoli	Cauliflower
	*Mesophilic*											
T0	5.4 ± 0.2 bc	6.6 ± 0.1 a	6.7 ± 0.1 a	4.8 ± 0.2 cde	5.3 ± 0.2 bcd	5.6 ± 0.1 b	3.9 ± 0.2 fg	4.7 ± 0.1 de	4.5 ± 0.2 ef	3.0 ± 0.4 h	2.5 ± 0.4 h	3.7 ± 0.9 g
T1	7.7 ± 0.3 de	7.3 ± 0.2 ef	8.0 ± 0.2 cd	8.8 ± 0.1 b	8.5 ± 0.9 bc	9.4 ± 0.0 a	7.0 ± 0.0 f	7.0 ± 0.0 f	7.3 ± 0.2 ef	7.2 ± 0.3 ef	7.0 ± 0.0 f	7.5 ± 0.5 def
	***	***	***	***	***	***	***	***	***	***	***	***
	*Psychrophilic*											
T0	6.8 ± 0.1 a	6.8 ± 0.2 a	7.0 ± 0.1 a	4.9 ± 0.2 b	2.6 ± 0.6 c	4.7 ± 0.2 b	2.3 ± 0.3 c	1.6 ± 0.4 ef	1.9 ± 0.4 def	2.4 ± 0.3 cd	1.5 ± 0.2 f	2.2 ± 0.3 cde
T1	8.4 ± 0.2 d	7.8 ± 0.2 e	8.5 ± 0.2 cd	8.8 ± 0.1 bc	8.9 ± 0.1 b	9.6 ± 0.2 a	7.0 ± 0.0 f	7.0 ± 0.0 f	7.2 ± 0.2 f	7.2 ± 0.4 f	7.0 ± 0.0 f	7.2 ± 0.3 f
	***	***	***	***	***	***	***	***	***	***	***	***
	*Molds and yeasts*											
T0	5.2 ± 0.1 a	5.1 ± 0.1 a	5.5 ± 0.1 a	1.0 ± 0.0 c	1.1 ± 0.1 bc	1.6 ± 0.6 b	1.3 ± 0.2 b	1.3 ± 0.2 bc	1.0 ± 0.0 c	1.1 ± 0.1 bc	1.2 ± 0.2 bc	1.2 ± 0.4 bc
T1	5.6 ± 0.2 a	4.5 ± 0.3 b	4.6 ± 0.4 b	4.3 ± 0.5 b	4.1 ± 0.1 b	4.2 ± 0.6 b	4.0 ± 0.0 b	4.2 ± 0.3 b	4.0 ± 0.0 b	4.0 ± 0.0 b	4.0 ± 0.0 b	4.7 ± 0.9 b
	*	**	**	***	***	***	***	***	ns	***	***	***
	*Coliforms*											
T0	3.0 ± 0.1 c	2.8 ± 0.2 c	4.8 ± 0.2 a	2.1 ± 0.3 d	1.5 ± 0.2 e	4.0 ± 0.1 b	1.0 ± 0.0 f	1.0 ± 0.0 f	1.9 ± 0.4 def	1.0 ± 0.0 f	1.0 ± 0.0 f	1.1 ± 0.1 f
T1	4.3 ± 0.3 b	4.2 ± 0.2 bc	5.0 ± 0.2 a	4.0 ± 0.0 c	4.0 ± 0.0 c	4.0 ± 0.0 c	4.0 ± 0.0 c	4.0 ± 0.0 c	4.0 ± 0.0 c	4.0 ± 0.0 c	4.0 ± 0.0 c	4.0 ± 0.0 c
	***	***	ns	***	***	ns	ns	ns	***	ns	ns	***
	*S. aureus*											
T0	2.9 ± 1.3	2.5 ± 0.3	2.6 ± 0.3	2.0 ± 0.0	2.1 ± 0.1	2.1 ± 0.1	2.0 ± 0.0	2.0 ± 0.0	2.0 ± 0.0	2.0 ± 0.0	2.0 ± 0.0	2.0 ± 0.0
T1	2.7 ± 0.4	2.2 ± 0.3	2.1 ± 0.1	2.6 ± 0.5	2.0 ± 0.0	2.1 ± 0.1	2.4 ± 0.5	2.0 ± 0.0	2.0 ± 0.0	2.2 ± 0.2	2.0 ± 0.0	2.0 ± 0.0
	ns	ns	*	ns	ns	ns	ns	ns	ns	ns	ns	ns
	*Clostridium*											
T0	0.0 ± 0.0 b	0.0 ± 0.0 b	1.0 ± 0.0 a	0.2 ± 0.4 b	0.0 ± 0.0 b	0.2 ± 0.4 b	0.0 ± 0.0 b	0.4 ± 0.5 b	0.2 ± 0.4 b	0.0 ± 0.0 b	0.0 ± 0.0 b	0.0 ± 0.0 b
T1	0.0 ± 0.0 a	0.0 ± 0.0 b	0.4 ± 0.5 b	0.0 ± 0.0 ab	0.0 ± 0.0 b	0.6 ± 0.5 b	0.0 ± 0.0 a	0.0 ± 0.0 b	0.2 ± 0.4 b	0.0 ± 0.0 b	0.0 ± 0.0 b	0.0 ± 0.0 b
	ns	ns	ns	ns	ns	ns	ns	ns	ns	ns	ns	ns

T0: 1 day of storage T1: 14 days of storage. Broccoli: lamb burgers wit 5% of broccoli by-product. Cauliflower: lamb burgers wit 5% of cauliflower by-product. HHP1: 400 MPa 60 s; HHP2: 500 MPa 60 s; HHP3: 600 MPa 60 s. Means values followed by different letters indicate the existence of significant differences between treatments by the Tukey test (*p* ≤ 0.05). ns indicates no difference; * Significance at *p* ≤ 0.05; ** Significance at *p* ≤ 0.01; *** Significance at *p* ≤ 0.001.

**Table 6:** Effect of the incorporation of the acidified *Brassica* by product ingredients in treated HPP and non-treated burgers.

		Non-Treated			HHP3		
		Lamb	Broccoli	Cauliflower	Lamb	Broccoli	Cauliflower
*pH*	T0	5.83 ± 0.1 b	5.85± 0.1 b	5.8 ± 0.1 b	6.0 ± 0.1 a	6.0 ± 0.1 a	6.0 ± 0.2 a
*a_w_*	T0	0.926 ± 0.001	0.929 ± 0.001	0.929 ± 0.002	0.929 ± 0.002	0.928 ± 0.002	0.925 ± 0.002
*Instrumental color*							
CIE L*	T0	55.2 ± 0.8 b	55.0 ± 0.4 b	55.1 ± 0.8 b	62.2 ± 0.6 a	62.7 ± 0.7 a	63.1 ± 0.3 a
	T1	57.9 ± 0.8 b	56.7 ± 0.5 c	57.9 ± 0.9 b	61.2 ± 0.6 a	62.0 ± 0.6 a	62.3 ± 0.3 a
		***	***	**	ns	ns	ns
CIE a*	T0	8.0 ± 0.5 a	7.9 ± 0.4 a	7.7 ± 0.2 a	2.4 ± 0.1 b	2.7 ± 0.1 b	2.6 ± 0.2 b
	T1	5.9 ± 0.3 ab	6.2 ± 0.3 a	5.5 ± 0.3 b	2.3 ± 0.4 c	2.4 ± 0.4 c	2.8 ± 0.2 c
		***	***	***	ns	ns	ns
CIE b*	T0	11.8 ± 0.4 ab	12.3 ± 0.4 ab	12.6 ± 0.6 a	11.3 ± 0.4 b	12.0 ± 0.5 ab	11.9 ± 0.6 ab
	T1	10.3 ± 0.3 d	10.7 ± 0.1 cd	10.9 ± 0.3 c	11.3 ± 0.2 b	11.8 ± 0.5 ab	11.8 ± 0.3 a
		***	**	**	ns	ns	ns
*Oxidation*							
*TBA-RS* *(mg kg^−1^)*	T0	0.4 ± 0.1 b	0.4 ± 0.1 b	0.4 ± 0.1 b	0.6 ± 0.1 ab	0.5 ± 0.1 ab	0.6 ± 0.0 a
	T1	0.9 ± 0.0 a	1.0 ± 0.0 a	1.0 ± 0.0 a	0.7 ± 0.1 b	0.7 ± 0.2 b	0.7 ± 0.2 b
		***	***	***	ns	ns	ns
*Carbonyls* *(Nmol mg^−1^ protein)*	T0	1.5 ± 0.2 b	1.6 ± 0.2 b	2.7 ± 0.2 a	2.6 ± 0.6 ab	2.2 ± 0.5 ab	2.9 ± 0.5 a
	T1	26.9 ± 4.6 c	32.8 ± 2.8 b	44.8 ± 9.4 a	2.4 ± 0.5 d	3.9 ± 0.9 d	3.1 ± 1.1 d
		***	***	***	ns	*	ns
*Microbial counts (log CFU g^−1^)*							
*Mesophilic*	T0	7.3 ± 0.1 a	7.1 ± 0.1 a	7.2 ± 0.1 a	3.7 ± 0.1 b	3.7 ± 0.1 b	3.8 ± 0.1 b
	T1	7.8 ± 0.3 a	7.3 ± 0.1 ab	7.3 ± 0.1 ab	5.1 ± 1.0 b	4.7 ± 1.2 b	6.1 ± 0.7 ab
		*	**	ns	*	ns	**
*Psychrophilic*	T0	7.3 ± 0.1 a	7.1 ± 0.1 a	7.2 ± 0.1 a	2.1 ± 0.2 b	2.3 ± 0.3 b	2.1 ± 0.1 b
	T1	8.1 ± 0.1 a	8.0 ± 0.1 a	8.1 ± 0.1 a	5.0 ± 1.4 b	4.6 ± 1.3 b	6.1 ± 0.5 b
		**	***	***	*	*	**
*Molds and yeasts*	T0	4.7 ± 0.1 a	4.6 ± 0.1 a	4.7 ± 0.2 a	1.3 ± 0.4 c	2.7 ± 1.0 b	2.3 ± 1.1 bc
	T1	4.5 ± 0.6 a	3.2 ± 0.2 ab	3.1 ± 0.3 ab	2.0 ± 0.9 b	2.0 ± 1.3 bc	2.2 ± 1.5 bd
		ns	***	***	ns	ns	ns
*Coliforms*	T0	2.1 ± 0.1 a	1.5 ± 0.6 ab	2.1 ± 0.2 a	ND b	ND bc	ND bd
	T1	1.3 ± 0.3	1.30 ± 0.5	1.1 ± 0.1	ND	ND	ND
		***	ns	***	ns	ns	ns
*S. aureus*	T0	2.4 ± 0.3 b	2.9 ± 0.1 a	3.0 ± 0.2 a	2.1 ± 0.1 c	ND c	ND cd
	T1	2.0 ± 0.0 a	2.1 ± 0.1 a	2.1 ± 0.1 a	2.2 ± 0.4 ab	2.7 ± 0.5 b	2.4 ± 0.3 ab
		ns	***	***	ns	*	ns
*Clostridium*	T0	1.1 ± 0.3	ND	ND	ND	ND	ND
	T1	ND	ND	ND	ND	ND	ND
		ns	ns	ns	ns	ns	ns
*Sensory analysis*							
*Appearance*	T0	7.4 ± 1.0	7.3 ± 1.2	7.8 ± 1.1	7.4 ± 1.4	7.3 ± 1.6	7.4 ± 1.2
*Odor*	T0	7.3 ± 1.1	7.1 ± 1.4	7.1 ± 1.4	7.2 ± 1.4	7.0 ± 1.3	7.2 ± 1.2
*Taste*	T0	6.5 ± 1.3	7.0 ± 1.5	7.0 ± 1.5	7.2 ± 1.6	7.0 ± 1.6	7.3 ± 1.3
*Texture*	T0	7.0 ± 1.3	7.1 ± 1.5	7.2 ± 1.6	6.8 ± 1.8	6.4 ± 1.9	6.7 ± 1.0
*Unpleasant taste*	T0	0.1 ± 0.2	0.2 ± 0.3	0.2 ± 0.3	0.1 ± 0.2	0.1 ± 0.2	0.1 ± 0.1
*Global Evaluation*	T0	7.1 ± 1.2	7.1 ± 1.5	7.2 ± 1.4	7.1 ± 1.7	7.1 ± 1.6	7.2 ± 1.1

T0: 1 day of storage T1: 14 days of storage. Broccoli: lamb burgers with 5% of broccoli by-product. Cauliflower: lamb burgers with 5% of cauliflower by-product. HHP3: 600 MPa 60 s. Mean values followed by different letters indicate the existence of significant differences between treatments by the Tukey test (*p* ≤ 0.05). ns indicates no difference; * Significance at *p* ≤ 0.05; ** Significance at *p* ≤ 0.01; *** Significance at *p* ≤ 0.001. ND: non-detected ≤1 or 2 log CFU g^−1^.

## Data Availability

The authors confirm that the data supporting the findings of this study are available within the article, and the raw data that support the findings are available from the corresponding author, upon reasonable request.

## Supplementary Materials

The following supporting information can be downloaded at: https://www.mdpi.com/article/10.3390/foods14040594/s1, Table S1: Fatty Acids Profile (FAP) of lamb burgers made with different ingredients (broccoli and cauliflower) and without any of them (Lamb).
