# Supplementary material for: Enhancing Shelf Life and Nutritional Quality of Lamb Burgers with Brassica By-Products: A Synergistic Approach Using High Hydrostatic Pressure
Source: Foods. 2025 Feb 11;14(4):594. doi: 10.3390/foods14040594 (PMC11853784; doi:10.3390/foods14040594)
Supplement: Supplementary file 1 [file foods-14-00594-s001.zip › foods-3398414-supplementary.pdf]

Table S1. Fatty Acids Profile (FAP) of lamb burgers made with different ingredients (broccoli and cauliflower) and without any of them (Lamb).

Burgers	Lamb	Broccoli	Cauliflower	*p-value*
**Fatty acids profile (%)**				
C12:0	0.44±0.01a	0.52±0.04a	0.32±0.01b	0.000
C14:0	4.71±0.08b	5.30±0.25a	4.15±0.1b	0.001
C16:0	24.59±0.15	24.78±0.13	24.76±0.16	0.609
C16:1	3.42±0.08a	2.81±0.03b	2.87±0.09b	0.000
C17:0	2.13±0.04a	1.65±0.02c	1.82±0.03b	0.000
C17:1	1.20±0.06a	0.71±0.02b	0.82±0.03b	0.000
C18:0	14.37±0.16b	17.97±0.18a	18.36±0.52a	0.000
C18:1	44.80±0.16a	41.66±0.33c	43.04±0.26b	0.000
C18:2 cis-cis	3.48±0.06a	3.43±0.11a	3.01±0.03b	0.001
C18:3	0.74±0.03a	0.10±0.06b	0.70±0.02a	0.001
C20:0	0.030±0.00	0.038±0.01	0.04±0.01	0.315
C20:1	0.09±0.00b	0.12±0.01a	0.10±0.01b	0.025
SFA	44.14±0.54b	48.61±0.86a	47.64±0.77a	0.000
MUFA	51.64±0.60a	46.96±0.79c	48.65±0.79b	0.000
PUFA	4.22±0.08a	4.43±0.22a	3.71±0.07b	0.000

SFA: saturated fatty acids (sum of C12:0, C14:0, C16:0, C17:0, C18:0, C20:0); MUFA: monounsaturated fatty acids (sum of C16:1, C17:1, C18:1, C20:1); PUFA: polyunsaturated fatty acids (C18:2 cis-cis, C18:3). Means values followed by different letters indicate the existence of significant differences between treatments by the Tukey test. Significant at p≤0.05; very significant p≤0.01; highly significant at p≤0.001.
